# Detection of IgA and IgG Antibodies against the Structural Proteins of SARS-CoV-2 in Breast Milk and Serum Samples Derived from Breastfeeding Mothers

**DOI:** 10.3390/v15040966

**Published:** 2023-04-14

**Authors:** Karen Cortés-Sarabia, Vianey Guzman-Silva, Karla Montserrat Martinez-Pacheco, Jesús Alberto Meza-Hernández, Víctor Manuel Luna-Pineda, Marco Antonio Leyva-Vázquez, Amalia Vences-Velázquez, Fredy Omar Beltrán-Anaya, Oscar Del Moral-Hernández, Berenice Illades-Aguiar

**Affiliations:** 1 Laboratorio de Inmunobiología y Diagnóstico Molecular, Facultad de Ciencias Químico Biológicas, Universidad Autónoma de Guerrero, Chilpancingo de los Bravo 39086, Mexico; 2 Unidad de Investigación en Inmunología y Proteómica, Laboratorio de Investigación en COVID-19, Hospital Infantil de México “Federico Gómez”, Mexico City 06720, Mexico; 3 Laboratorio de Biomedicina Molecular, Facultad de Ciencias Químico Biológicas, Universidad Autónoma de Guerrero, Chilpancingo de los Bravo 39086, Mexico; 4 Laboratorio de Virología, Facultad de Ciencias Químico Biológicas, Universidad Autónoma de Guerrero, Chilpancingo de los Bravo 39086, Mexico

**Keywords:** antibodies, IgA, IgG, breastfeeding, COVID-19, structural proteins, SARS-CoV-2

## Abstract

**Background:** COVID-19 vaccination or natural infection is associated with the development of immunity. The search of IgA and IgG antibodies against all the structural proteins (spike, nucleocapsid, membrane, and envelope) of SARS-CoV-2 in breastfeeding mothers is associated with immunity that can help the newborn avoid development of the infection. **Methods:** In this study, we analyzed 30 breastfeeding women that provided samples of breast milk and serum and evaluated the presence of IgA, total IgG, and subclasses against the structural proteins of SARS-CoV-2. **Results:** We reported a high seroprevalence to IgA (76.67–100%) and negativity to IgG against all analyzed proteins in breast milk. Seroprevalence in serum samples was around 10–36.67% to IgA and 23.3–60% to IgG. Finally, we detected the presence of the subclasses IgG1, IgG2, and IgG4 against all the structural proteins of SARS-CoV-2. **Conclusions:** This work provides evidence of the presence of IgA and IgG antibodies against the four structural proteins of SARS-CoV-2 in breast milk and serum samples derived from breastfeeding women, which can confer immunity to the newborn.

## 1. Introduction

COVID-19, or coronavirus disease 2019, is highly contagious and affects the human respiratory system. The incubation period of COVID-19 is 5 to 7 days [1] and includes symptoms such as cough, fever, dyspnea, fatigue, and others [2]. COVID-19 promotes severe pulmonary damage and progressive respiratory failure. This disease is associated with infection by SARS-CoV-2, which is transmitted to humans by exposure to infectious respiratory fluids and fomites [3,4]. SARS-CoV-2 has a spherical shape with a diameter of around 80–120 nm, contains spike-like structures, has a single-stranded positive-sense RNA (+RNA) genome that encodes for accessory proteins (ORF; open read frames), 16 non-structural proteins (nsp1–16), and four structural proteins (S, N, M, and E) associated with the formation of the viral particle [5,6,7]. The spike protein (S) has 1273 amino acids and two sub-units (S1 and S2); its primary function is binding the virus with the host cell. The nucleocapsid protein (N) has 419 amino acids and two domains (RNA-binding domain and dimerization domain), allowing for the packing of the viral RNA. The membrane protein (M) has 222 amino acids and is the most abundant structural protein; it contains three structural domains and is the only protein that interacts with all the structural proteins. Finally, the envelope protein (E) has 75 amino acids and is involved in the viral cycle (assembly, budding, envelope formation, and pathogenesis) [8,9].

Vaccination against COVID-19 in women during pregnancy or breastfeeding is essential to providing immunity to the newborn and increasing the available data about the effectiveness of vaccines in this specific risk group [10]. Breastfeeding is an exclusive characteristic of mammals and consists of the secretion of milk from the mammary glands with a high nutritional value that allows for development and confers protection against several diseases during the first weeks or months after birth [11,12]. The conferred protection of breast milk is associated with the presence of IgA and IgG capable of recognizing proteins of SARS-CoV-2 [13]. Previous studies have described the presence of antibodies against SARS-CoV-2 in breast milk and serum samples derived from breastfeeding women either with natural infection or who are vaccinated and have reported a correlation between the effectivity of vaccination and the neutralizing activity of antibodies [14,15,16]. IgA and IgG antibodies in breast milk and serum samples of breastfeeding women were associated with protecting the newborn against the transmission or the development of severe COVID-19 [17]. To our knowledge, there are only a few studies describing the presence of antibodies against all the structural proteins of SARS-CoV-2 in breastfeeding women, and this communication aimed to provide evidence of the presence of IgA and IgG against S, N, M, and E proteins of SARS-CoV-2 in breast milk and serum samples derived from naturally infected or vaccinated breastfeeding women.

## 2. Materials and Methods

### 2.1. Study Design and Participants

We conducted an observational study aimed at analyzing the presence of IgA and IgG antibodies against all the structural proteins of SARS-CoV-2 in breast milk and serum samples derived from 30 vaccinated/naturally infected breastfeeding mothers. Samples were collected from October to December 2022. The inclusion criteria were: COVID-19 vaccination (at least one dose) or natural infection by SARS-CoV-2, breastfeeding (exclusive or mixed), and a number of children (single child or more). A survey was used for the collection of clinical and epidemiological data. It included: age; diagnosis of COVID-19 via RT-PCR, antigen, or clinical/unknown (not reported by the patient); serological status; symptomatology (fever, headache, loss of smell, loss of taste, dyspnea, chest pain, cough, sore throat, burning eyes, congested nose, muscle pain, joint pain, fatigue, chills, vomit, and diarrhea); number of infections; vaccine doses; and type of breastfeeding.

### 2.2. Collection of Samples

Lactating mothers provided breast milk and serum samples. The breast milk was self-collected, manually or by breast pumping in sterile conical tubes. Later, a blood sample was obtained by venipuncture by trained personnel. We centrifuged the blood sample and placed the serum in a new sterile tube. Collected samples were immediately processed and remains were stored at −20 °C for additional analysis.

### 2.3. IgA and IgG Antibody Detection (Indirect ELISA)

As an antigen, we used recombinant proteins derived from the SARS-CoV-2 (S1 domain, RBD domain; Receptor Binding Domain, N, M, and E), and purification was performed as previously described [18]. Microtiter plates (Sigma-Aldrich, St. Louis, MO, USA) were coated with 100 µL/well of each antigen (S1 domain, RBD domain, N, M, and E) to a final concentration of 0.1 µg/mL in a coating buffer (50 mM Na_2_CO_3_/NaHCO_3_, pH 9.6). The plates were incubated for one hour at 37 °C and then blocked for 40 min at 37 °C with 200 µL/well of 5% skimmed milk diluted in phosphate-buffered saline (PBS)-Tween 20 (0.05%). For the primary antibody, 100 µL/well of serum samples (1:50 diluted in PBS) or breast milk (direct) by duplicate were incubated for one hour at 37 °C. Finally, plates were incubated with 100 µL/well of monoclonal anti-human IgA (Sigma-Aldrich; 1:500 dilution) and IgG (Sigma-Aldrich; 1:1500 dilution, St. Louis, MO, USA) coupled to horseradish peroxidase (HRP) for one hour at 37 °C. After every step, the plates were washed thrice with 200 μL/well of PBS-Tween 20 0.05% for 5 min. The enzymatic reaction was developed using o-phenylenediamine dihydrochloride (Sigma-Aldrich) and stopped using 2 N H_2_SO_4_. The optical density (OD) was measured at 492 nm using a microplate reader. Samples with OD > 0.300 were considered positive for both antibodies.

### 2.4. Anti-IgG Subclass Detection

For IgG subclass evaluation, antigens were fixed and plates were blocked as described above. Serum samples were prepared (1:25 diluted in PBS), and each sample was tested by duplicate to each antigen and subclass. Plates were incubated for 30 min at 37 °C. Later, monoclonal antibodies against each subclass: IgG1 (1:500; Sigma-Aldrich Cat# B6775), IgG2 (1:25,000; Sigma-Aldrich Cat#B3398), IgG3 (1:500; Sigma-Aldrich Cat#B3523), and IgG4 (1:1,000; Sigma-Aldrich Cat#B3648) were added for 45 min at 37 °C. As a secondary antibody, anti-mouse IgG (H+L) coupled to HRP (Invitrogen Cat# 62-6520) was diluted 1:11,000 for 45 min at 37 °C. After each step, plates were washed for 7 min with PBS-Tween 20 0.05%. Finally, the enzymatic reaction was developed as above. The cut-off value for each antibody was: IgG1 ≥ 0.070, IgG2 ≥ 0.175, IgG3 ≥ 0.112, and IgG4 ≥ 0.100, as we previously described (manuscript under review).

### 2.5. Statistical Analysis

A database was constructed in STATA V.16 using all analyzed variables and results from each evaluation. For statistical analysis, we used Fisher’s exact test, Chi-square test, and Kruskal–Wallis, and a *p*-value < 0.05 was considered statistically significant. The heat maps and graphs were constructed using GraphPad Prism V.8.

### 2.6. Ethics Statement

Breastfeeding mothers were invited to participate in this study, and sample collection was performed after signing informed consent according to the Helsinki declaration. All results were treated with confidentiality, and this study was approved by the ethics committee of the Universidad Autonóma de Guerrero (CB-003/22).

## 3. Results

### 3.1. Characteristics of the Population

Serum and breast milk samples were collected from 30 breastfeeding mothers. A survey was used for clinical and epidemiological data collection. Participants were 18–39 years old, and most (67%) were first-time mothers. The primary delivery method was cesarean section (70%), and 77% of newborns were a mature term with average weight (76.67%). Mothers reported performing mainly exclusive (67%) breastfeeding, and, for this study, they provided transitional or mature milk (93.3%) (Table 1).

**Figure 1:**
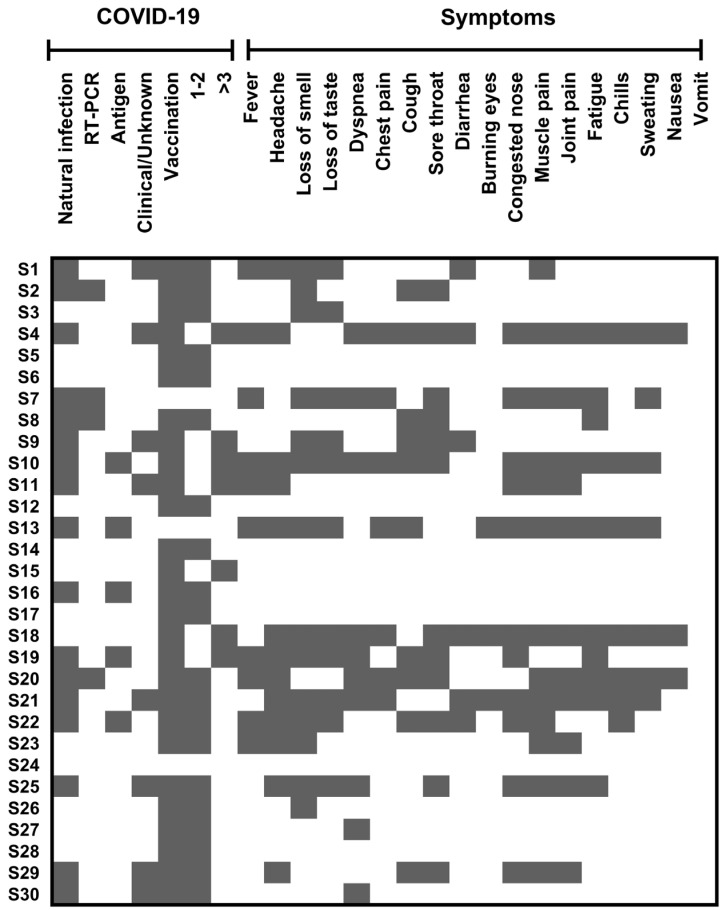
**COVID-19 and related symptoms in the studied population.** Heat map representing natural infection, diagnostic (RT-PCR, antigen, clinical/unknown), vaccination, number of doses (1–2 or ≥3), and symptoms related to the infection. S: sample and 1–30: designed number for each participant. Grey parts indicate the presence of the analyzed variable.

Concerning natural infection and vaccination against COVID-19, 56.7% were infected by SARS-CoV-2; the employed diagnostic method included RT-PCR (13.3%), antigen testing (16.7%), and clinical/unknown methods (26.6%). Elapsed time since the last infection was <6 months in 54% of the cases. Regarding vaccination, 90% of patients received at least 1–2 doses (67%) or ≥3 (23%). The primary type of applied vaccines was: AstraZeneca (23.3%), Pfizer (33.3%), and Sinovac (23.3%), whereas the booster was homologous (administration of the same vaccine) and heterologous (booster that does not match the primary vaccination) in 46.7% and 43.3% of cases, respectively. The elapsed time since the last boost in most of the cases was ≥6 months (66.7%) (Table S1). The main symptoms were fever, chest pain, diarrhea, burning eyes, chills, sweating, and nausea (Figure 1).

### 3.2. IgA and IgG Antibody Detection in Breast Milk and Serum Samples of Breastfeeding Women

**Figure 2:**
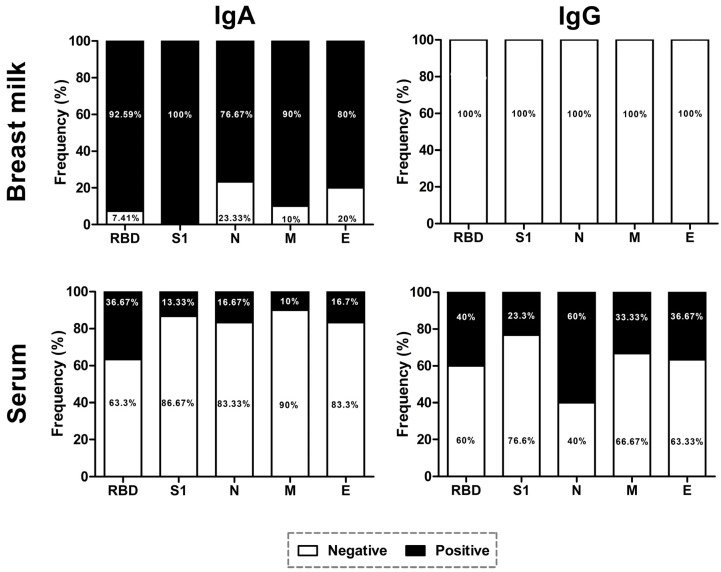
**IgA and IgG seroprevalence against structural proteins of SARS-CoV-2 in breast milk and serum samples.** Collected samples derived from breastfeeding women were tested against the recombinant RBD and S1 domain of the spike protein, nucleocapsid, membrane, and envelope of the SARS-CoV-2.

We evaluated the presence of IgA and IgG antibodies against each structural protein of SARS-CoV-2; particularly for the S protein, we used recombinant antigens derived from the S1 domain and RBD for the specific detection of antibodies in the N-terminal domain and glycosylated parts. In breast milk, IgA antibodies against the S1 domain were detected in 100% of the samples, 92.59% for the RBD. In relation to N, M, and E, the prevalence was 76.67%, 90%, and 80%, respectively. For IgG detection in breast milk, all samples tested negative. For the detection of IgA in serum samples, only 36.67% tested positive for the RBD, 13.33% for S1, 16.67% for N, 10% for M, and 16.7% for E. Finally, the detection of IgG in serum samples was as follows: 40% were positive to RBD; 23.3% to S1; 60% to N; 33.33% to M; and 36.67% to E (Figure 2). Later, samples were classified according to positivity only to RBD/S1, positivity to N/M/E, or positivity to antibodies in the S protein and another structural protein to analyze the presence of antibodies related only to vaccination (RBD/S1) or symptomatic or asymptomatic natural infection (N/M/E or RBD/S1/N/M/E). In breast milk, two samples were positive for RBD/S1, three were positive for N/M/E, and 25 tested positive for antibodies in RBD/S1/N/M/E. In serum samples, 17 were negative for IgA, five were positive for RBD/S1, two were positive for N/M/E, and six were positive for RBD/S1/N/M/E. For the detection of IgG in serum samples, four were negative, four tested positive for RBD/S1, seven were positive for N/M/E, and 15 were positive for RBD/S1/N/M/E (Figure S1).

**Figure 3:**
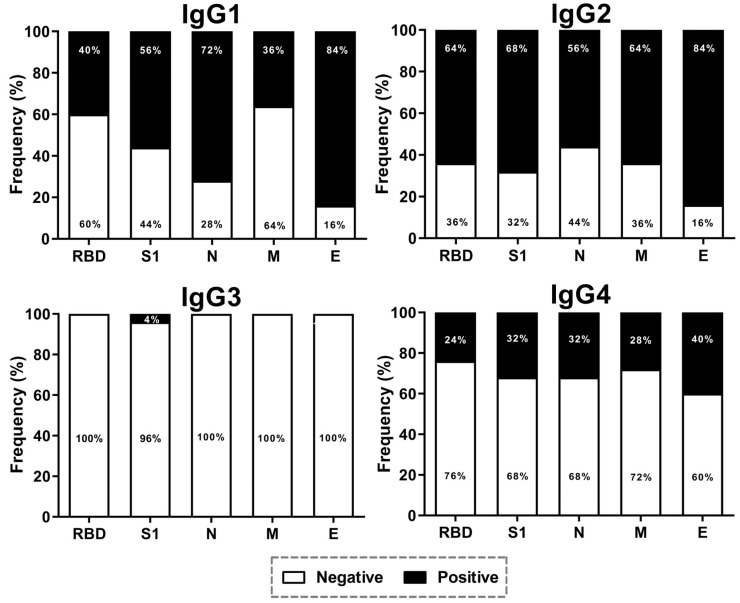
**IgG subclasses in serum samples.** IgG subclass detection in the 26 positive samples; this protocol included the use of monoclonal antibodies against each subclass (produced in mouse) and an antibody anti-mouse coupled to HRP.

Lastly, we evaluated the presence of IgG subclasses in the 25 positive serum samples. In relation to IgG1: 40% of samples were positive for RBD, 56% for S1, 72% for N, 36% for M, and 84% for E. IgG2: 64% were positive for RBD, 68% for S1, 56% for N, 64% for M, and 84% for E. In IgG3, only 4% were positive for S1. Finally, for IgG4: 24% were positive for RBD, 32% for S1, 32% for N, 28% for M, and 40% for E (Figure 3).

## 4. Discussion

COVID-19 disease during pregnancy can lead to severe disease and vertical transmission to the fetus [19]; thus, pregnant women are encouraged to be vaccinated. The development of immunity in the newborn is associated with placental transmission of IgG during pregnancy and the transmission of immune factors by breast milk (chemokines, cytokines, immunoglobulins, hormones, growth factors, lymphocytes, neutrophils, and macrophages) [20,21]. Breastfeeding provides health benefits for the child and mother; the support of breastfeeding begins during pregnancy and continues through the early life of the child [22]. This study aimed to search for IgG and IgA antibodies in blood samples and breast milk against all the structural proteins of SARS-CoV-2 in breastfeeding women. Our studied population was integrated by 30 breastfeeding women between 18–39 years old, of which 70% had delivery via cesarean section. The type of delivery is associated with the maturation, structure, and function of the immune system, and the composition of the microbiota of the newborn delivered by cesarean is less diverse compared to the bacterial species found in the newborn delivered vaginally [23].

Newborns were mature term (77%) with average weight (76.77%), and most of the mothers performed exclusive (67%) breastfeeding. The gestational age is associated with disease risk, growth, weight, and asthma development. Premature newborns (32–36 weeks) have higher neonatal morbidity and mortality in comparison to newborns of 37 or more weeks [24]. Exclusive breastfeeding is essential for the newborn’s survival, growth, development, health, and nutrition. It confers protection against infectious disease and contains immunomodulatory, anti-inflammatory and antimicrobial factors, that help to reduce mortality risk in the short and long term and influence the cognitive and psychomotor learning of the child in comparison with those that consume only formula or mixed breastfeeding [25]. Concerning natural infection and vaccination against COVID-19, 56.7% reported natural infection, and 90% were vaccinated with at least one dose; the time elapsed since the last booster is >6 months (66.7%). Pregnant women are more susceptible to the acquisition of SARS-CoV and to developing severe COVID-19 than non-pregnant women. In contrast, vaccination helps to reduce morbidity and mortality worldwide to infectious diseases [26]. COVID-19 infection or vaccination generates antibodies against the structural antigens of SARS-CoV-2, which have several biological activities, such as neutralization, antibody-dependent phagocytosis (ADCP), complement, and NK cell activation [27,28].

In this work, we reported a high prevalence of IgA antibodies in breast milk against the S (RBD; 92.59% and S1; 100%), N (76.67%), M (90%), and E (80%). IgA is mainly found in breast milk, representing around 90% of the total immunoglobulins [29]. The biological functions of the SIgA include neutralizing intracellular viruses and the inhibition of the pathogen to the host mucosa and the agglutination of bacteria and viruses [30]. Several studies have been performed to evaluate the presence of IgA antibodies in the breast milk of vaccinated/naturally infected women against COVID-19. Pearl et al. reported a high secretion of neutralizing IgA and IgG in breast milk six weeks after vaccination with mRNA vaccines [31]. In contrast, other studies have reported the presence of IgA anti-RDB in breast milk [27,32] and provided evidence of the in vitro capacity of IgA antibodies (derived from natural infection) to neutralize SARS-CoV-2 infectivity [33]. With regards to IgG in breast milk, all tested samples were negative, which could be associated with the absence of the secretory component in IgG and the capacity of this immunoglobulin to be transported through the epithelium. In addition, IgG is more dominant in colostrum [30], and the analyzed types of breast milk included transitional and mature milk. In serum samples, the prevalence of anti-RBD (40%) and anti-N (60%) was higher than the other analyzed structural proteins of SARS-CoV-2. After vaccination, it has been reported that IgG is the predominant antibody, and its production continues six months after vaccination [34,35].

The detection of IgA and IgG antibodies against N, M, and E are associated with the symptomatic or asymptomatic infection of SARS-CoV-2. The N protein participates in the RNA package and virus release. The presence of IgA, IgM, and IgG antibodies against the N antigen in COVID-19 patients has been confirmed by immunoblot assays [36]. The M protein is the most abundant protein of SARS-CoV-2 and contributes to viral infection; the presence of IgM and IgG antibodies against this protein has been reported in COVID-19 convalescent patients, thus providing evidence of the potential utility of M protein as an immunodominant antigen [37,38]. Finally, the E protein promotes the packaging and reproduction of the virus [39]. Anti-E antibodies have been reported in SARS-CoV-2 infected patients and healthy volunteers and can be produced during asymptomatic infection [40].

There are four subclasses of IgG antibodies, and they have a homology of 90%. The structure of the constant regions and biological functions defines the differences. IgG1 and IgG3 are produced mainly during viral infections [41], and the specific presence of IgG3 against the N protein and IgG1, IgG2, and IgG4 against the RBD has been reported [42]. The different antibody subclasses associated with natural infection or vaccination could recruit and activate innate immune cells that carry IgG receptors on the surface [43]. The production of IgG subclasses and IgA antibodies are allowed by somatic hypermutation. In addition, differences in the reported seroprevalence to each structural protein in breast milk and serum could be associated with the lifetime of plasma cells. Further studies need to assess the importance of the BCR repertory in the generation of diversity during SARS-CoV-2 infection or vaccination, as well as the presence of circulating plasma cells/memory cells and its association with IgG and IgA antibody levels.

## 5. Conclusions

The natural infection or vaccination of COVID-19 during pregnancy or breastfeeding is vital for developing immunity in the newborn. We reported a high prevalence of IgA antibodies against all the structural proteins of SARS-CoV-2 in breast milk (76.67–100%) and a moderate prevalence of IgG (23.3–60%) and IgA (10–36.67%) in serum samples. Our results suggest that breast milk contains antibodies capable of recognizing and neutralizing SARS-CoV-2 to confer immunity to the newborn. In the bloodstream, IgG is the most dominant, and its biological functions—associated with the specific presence of IgG1, IgG2, and IgG4 subclasses—are neutralization, complement activation, and phagocytosis, among others. It is imperative to perform evaluations focused on the presence of total IgG and IgA antibodies against the S and N proteins. However, we must also include M, E, and IgG subclasses to fully understand the immune response developed against SARS-CoV-2.

## Figures and Tables

**Table 1:** Characteristics of the studied population.

Variables	*n* (%)
**Age (years)**	
18–22	10 (33)
23–27	8 (27)
28–39	12 (40)
**Number of pregnancies**	
1	20 (67)
2	10 (33)
**Type of delivery**	
Natural	9 (30)
Cesarean section	21 (70)
**Gestational age**	
Early or immature term	6 (20)
Mature term	23 (77)
Post-term	1 (3)
**Newborn weight at birth**	
Low weight (1.500–2.500 kg)	6 (20)
Average weight (2.599–3.800 kg)	23 (76.67)
Overweight (3.800–4.000 kg)	1 (3.33)
**Breastfeeding type**	
Exclusive	20 (67)
Mixed	10 (33)
**Type of breast milk**	
Colostrum	2 (6.7)
Transitional milk	12 (40)
Mature milk	16 (53.3)
**Total**	30 (100)

## Data Availability

Data is contained within the article or Supplementary Material.

## Supplementary Materials

The following supporting information can be downloaded at: https://www.mdpi.com/article/10.3390/v15040966/s1, Table S1: COVID-19 natural infection and vaccination in breastfeeding women. Figure S1: Grouped IgA and IgG seroprevalence against the structural proteins of SARS-CoV-2. Heat map representing IgA and IgG evaluation results in serum samples and breast milk. The results were classified as: negative, positive only to RBD or S (vaccination), positive to N/M/E (natural infection), and RBD/S1 and at least one structural protein (RBD/S1/N/M/E) (natural infection). S: sample and 1–30: designed number to each participant. Grey parts indicate a positive result.
