# Supplementary material for: Detection of IgA and IgG Antibodies against the Structural Proteins of SARS-CoV-2 in Breast Milk and Serum Samples Derived from Breastfeeding Mothers
Source: Viruses. 2023 Apr 14;15(4):966. doi: 10.3390/v15040966 (PMC10144911; doi:10.3390/v15040966)
Supplement: Supplementary file 1 [file viruses-15-00966-s001.zip › Figure S1.pdf]

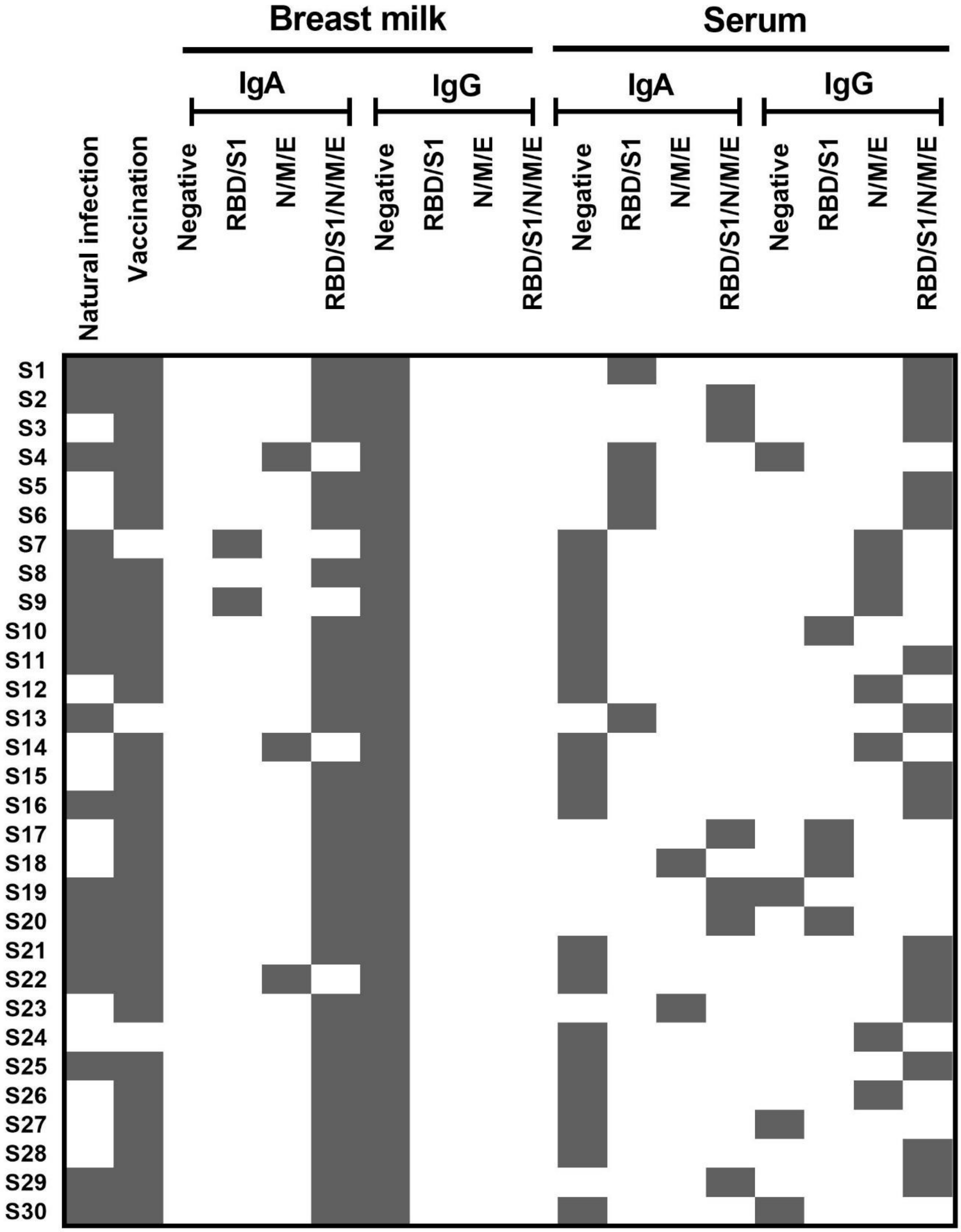

Figure S1.- Grouped IgA and IgG seroprevalence against the structural proteins of SARS-CoV-2. Heat map representing IgA and IgG evaluation results in serum samples and breast milk. The results were classified as: negative, positive only to RBD or S (vaccination), positive to N/M/E (natural infection), and RBD/S1 and at least one structural protein (RBD/S1/N/M/E) (natural infection). S: sample and 1-30: designed number to each participant. Grey parts indicate a positive result.
