# Supplementary material for: Detection of IgA and IgG Antibodies against the Structural Proteins of SARS-CoV-2 in Breast Milk and Serum Samples Derived from Breastfeeding Mothers
Source: Viruses. 2023 Apr 14;15(4):966. doi: 10.3390/v15040966 (PMC10144911; doi:10.3390/v15040966)
Supplement: Supplementary file 1 [file viruses-15-00966-s001.zip › Table S1.pdf]

Table S1. COVID-19 natural infection and vaccination in breastfeeding women.

Variables	n (%)
**COVID-19**	
No	13 (43.3)
Yes	17 (56.7)
**Type of diagnostic**	
None	13 (43.3)
RT-PCR	4 (13.3)
Antigen test	5 (16.7)
Clinical/Unknown	8 (26.6)
**Time since diagnosis**	
<6 meses	16 (54)
≥6 meses	14 (46)
**Vaccination**	
No	3 (10)
Yes	27 (90)
**Number of doses**	
None	3 (10)
1-2	20 (67)
>3	7 (23)
**Primary vaccination**	
None	3 (10)
AstraZeneca	7 (23.3)
Cansino	2 (6.7)
Pfizer	10 (33.3)
Sinovac	7 (23.3)
Other	1 (3.3)
**Booster Vaccination**	
None	3 (10)
Homologous	14 (46.7)
Heterologous	13 (43.3)
**Elapsed time since last boost**	
None	3 (10)
<6 months	7 (23.3)
≥6 months	20 (66.7)
**Total**	**30 (100)**
